# Instructors’ Gestural Accuracy Affects Geology Learning in Interaction with Students’ Spatial Skills

**DOI:** 10.3390/jintelligence11100192

**Published:** 2023-10-04

**Authors:** Corinne A. Bower, Lynn S. Liben

**Affiliations:** 1 Department of Psychology, California State University Los Angeles, Los Angeles, CA 90032, USA; 2 Department of Psychology, The Pennsylvania State University, University Park, PA 16802, USA; liben@psu.edu

**Keywords:** gestures, STEM learning, geology instruction, spatial skills, STEM education

## Abstract

Complex and often unobservable STEM constructs and processes are represented using a variety of representations, including iconic gestures in which the body is configured or moved to resemble a referent’s spatial properties or actions. Earlier researchers have suggested links between gesturing and expertise, leading some to recommend instructional gestures. Earlier research, however, has been largely correlational; furthermore, some gestures may be made with misleading positions or movements. Using the illustrative topic of strike in structural geology, we investigated the existence and impact of inaccurate instructional gestures. In Study 1, we examined videotapes of participants who had been asked to explain strikes to another person. We observed inaccurate (non-horizontal) strike gestures not only among novices (first introduced to strike during the study itself, n = 68) but also among participants who had greater expertise in geology (n = 21). In Study 2, we randomly assigned novices (N = 167) to watch video lessons in which the instructor accompanied verbal explanations of strikes with accurate, inaccurate, or no iconic gestures and tested students’ learning on a strike-mapping task. Students with low spatial-perception skills showed no impact of their gestural condition on performance. Students with high spatial-perception skills showed no advantage from accurate gestures but performed significantly worse in the inaccurate-gesture condition. Findings suggest that recommendations to use gestures during instruction should include professional development programs that reduce the occurrence of inaccurate gestures.

## 1. Introduction

Embodied cognition has become an increasingly common framework for theoretical and empirical work in domains such as cognitive development, cognitive neuroscience, spatial cognition, linguistics, and education. In this framework, bodily structures and processes are thought to affect not only individuals’ physical interactions with the external world but also their perceptual experiences and mental representations of that world (e.g., Glenberg 2008; Johnson 2007; Liben 2008; Tversky 2008). Thus, from the perspective of embodiment, representations used to process past experiences and visualize potential ones are thought to be grounded in individuals’ bodily structures and actions. An important branch of work that falls under the umbrella of embodied cognition concerns gestures in which external representations involving the body are used in the service of private thought or in the service of communicating ideas to others.

At the most general level, our program of research concerns ways that representations are created, used, and understood in educational contexts involving the disciplines of science, technology, engineering, and mathematics (STEM). The particular focus of the research described in the current paper addresses this topic by focusing on an illustrative concept relevant to a single STEM discipline—the concept of strike from structural geology—and on a particular type of representation—iconic gestures. These are gestures in which the body or a body part is oriented or moved to resemble a referent’s spatial properties or movements, as, for example, when one cups one’s hands together to make a spherical shape and then moves that shape in a circular path to represent the Earth’s orbit around the sun. In the remainder of the introduction, we explain the basis for our focus and review illustrative research on the production and impact of STEM-related representational gestures.

### 1.1. Why STEM?

We selected STEM as our focus for two reasons. First, it is a domain of practical importance that has been attracting increasing interest from—among others—educators, STEM scientists, labor economists, and cognitive-developmental psychologists. Success in STEM education is important in both national and global contexts for fulfilling labor needs, generating solutions to societal infrastructure and health needs, and advancing economic success (*Science and Engineering Indicators 2020: The State of U.S. Science and Engineering* 2020). It is important at the individual level because early preparation for STEM affects students’ ability and motivation to pursue a broad range of STEM educational and occupational opportunities in later life. 

Second, STEM fields include key concepts or phenomena that involve spatially challenging ideas. Spatial skills (e.g., mentally rotating or transforming objects and scenes) have been linked to students’ better understanding and application of concepts found in STEM fields such as physics (Hodgkiss et al. 2018), organic chemistry (Stull et al. 2012), astronomy (Bower and Liben 2020), and mathematics (e.g., Mix et al. 2016). Additionally, students who pursue STEM careers are more likely to have or develop stronger spatial skills than are those pursuing other careers (e.g., Wai et al. 2009). 

### 1.2. Why Geology in Particular?

Geology is an especially interesting STEM discipline to consider in relation to gesture because it is a highly spatial science (e.g., Atit et al. 2020; Kastens and Ishikawa 2006; Liben and Titus 2012) involving many phenomena that are not immediately visible to the eye (e.g., Earth’s interior structure, the rock cycle, geologic time). Among the concepts that are routinely taught in courses on structural geology are strike and dip, terms that refer to the orientation and inclination of rock outcrops, respectively. *Strike* is defined as “the direction of the line formed by the intersection of a fault, bed, or other planar feature and a horizontal plane” (Encyclopedia Britannica 1998). A strike line is drawn on a map to show the orientation of the outcrop. Strike is commonly expressed as degrees clockwise from true north (thus ranging between 0° and 360°, e.g., 047°); it can also be expressed as a compass bearing (e.g., N47° E). *Dip* refers to the direction and degree of inclination of the outcrop. On maps, a dip line is drawn perpendicular to the strike line; the side on which the dip line is drawn indicates the direction toward which the outcrop’s surface slopes downward. Written next to the dip line is the dip number, which indicates the steepness of the slope in degrees (between 0 and 90). Sample maps with strike and dip lines are included in Appendix A.2. Additional explanations about identifying strike orientation are provided in our overview of the current research (see Section 1.5). Given that STEM concepts like these are spatially challenging, it may be useful to think and communicate about them with the aid of external spatial representations, among which are gestures.

### 1.3. Iconic Gestures as STEM Representations

Most of the work on the creation, comprehension, and use of external spatial representations in STEM has involved 2- or 2½-dimensional graphic images such as maps or diagrams presented on paper or flat electronic displays (e.g., see National Research Council 2006). Our focus here, though, is on another kind of external spatial representation used to represent STEM concepts, objects, and processes—iconic gestures. This category of gestures refers to representations in which a body or body part is oriented or moved so that it resembles the spatial properties of the referential object or phenomenon in 3D space (e.g., an object’s shape or position), perhaps with movement to represent the referent’s changing structure or location over time. We ignore here gestures that do not convey spatial information about the referent via resemblance. The latter include *deictic* gestures, which draw attention to something for referential clarity (e.g., by pointing), and *beats*, which use body movements (e.g., arm movements) to reinforce the rhythm of speech (McNeill 1992).

Past work demonstrates associations between the use of iconic gestures and students’ STEM learning about phenomena that may or may not be directly visible in the physical world (Chue et al. 2015; Roth 2000, 2001). For example, iconic gestures have been associated with learning or reasoning in chemistry (e.g., Stieff et al. 2016), mechanical engineering (e.g., Hegarty et al. 2005), and geology (e.g., Atit et al. 2015; Kastens et al. 2008). Iconic gestures may be beneficial because they may allow the learner to offload storage of relevant premises to an external (gestural) representation, thereby freeing mental resources to solve the current task (Goldin-Meadow et al. 2001; Kastens et al. 2008; Weisberg and Newcombe 2017; Wesp et al. 2001). Spatial skills are linked to the use of representational gestures during STEM tasks or communications (DeSutter and Stieff 2017; Plummer et al. 2016; Weisberg and Newcombe 2017). In summary, past research has shown links between the use of iconic gestures and performance on spatially complex STEM concepts, including those in geology. 

An earlier investigation by Liben et al. (2010) focused explicitly on adults’ use of iconic gestures, while communicating about the geologic concepts of strike and dip. Individual participants with differing levels of prior expertise in structural geology were first asked to read a lesson about strike and dip. These participants were then asked to complete laboratory strike and dip tasks and, finally, to audio- and video-record explanations of strike and dip for another student who had been described as unfamiliar with structural geology. The investigators coded and analyzed participants’ gestures from recordings of the sessions. The data showed that participants across varying levels of expertise in structural geology commonly produced iconic gestures for their videotaped explanations. The data also revealed systematic links between some patterns of participants’ gesturing and their success on assessments of their own strike understanding. As reviewed next, other researchers have examined the link between STEM instructors’ use of gestures and their students’ performance on measures of STEM learning. 

### 1.4. Do Instructors’ Gestures Affect Learning? 

As just noted, some researchers have studied the impact of teachers’ gestures on student learning. For example, Alibali et al. (2013) provided one teacher with a tutorial on ways to use gestures during math instruction. The tutorial indeed led this teacher to increase his use of gestures during his subsequent instructional lesson. The teacher’s pre- and post-tutorial lessons were video-recorded, and samples of seventh-grade students were randomly assigned to watch one or the other videotape. A math test given after the lessons showed that learning was better among students who had been assigned to watch the teacher’s post-tutorial lesson, the lesson that included more gestures.

It is not only the presence and frequency of gestures that may affect students’ STEM learning—it is also the type of gesture. Suggesting that gesture types may have an impact on learning is research by Atit et al. (2016). They found that when instructors used pointing and tracing gestures to highlight contour lines on a topographic map, novices were better able to associate contour lines with elevation change. However, when instructors used three-dimensional iconic gestures and models, learning was not similarly enhanced. Most effective in leading students to extract three-dimensional shape information from topographic maps was the combination of a verbal emphasis on shape coupled with pointing and tracing gestures. 

Additionally, the accuracy of certain iconic gestures may influence student learning. For instance, if an instructor uses gestures that resemble the phenomenon being taught only weakly or perhaps even incorrectly, instructional gestures might confuse rather than aid the learner. Consistent with this point, when discussing the impact of strike gestures, Liben et al. (2010) noted the need for researchers to manipulate gestures systematically to test the impact of gestures on student learning. Similarly, in the concluding discussion of a review of the influence of gestures on learning, Goldin-Meadow (2014) suggested that it would be helpful for teachers to monitor their own gestures to avoid gestures that are potentially misleading. To our knowledge, however, there is no prior empirical research that experimentally manipulates the inclusion and accuracy of instructional gestures and then tests student learning.

### 1.5. Current Research

We designed our research to address two main questions. In Study 1, we sought to discover whether people who have been asked to explain the concept of strike to another person sometimes accompany their verbal explanations with iconic gestures that are spatially inconsistent with the meaning of their words. Our goal was to learn whether non-horizontal gestures appeared in the context of verbal descriptions that involved horizontality. For example, as illustrated by the dictionary definition of “strike” quoted above, many explanations of strike begin by referring to a line formed when the sloping face of an outcrop is intersected by a horizontal plane. Textbooks may illustrate this idea with a representational drawing of a sloping rock or hill adjacent to a still lake, which provides the intersecting horizontal plane, or by an abstract geometric drawing of a solid with a sloping face, which is intersected by a drawing of a horizontal plane. Several illustrative textbook figures are available online; see Liben et al. (2011). 

Importantly from the perspective of gesture, an instructor might accompany a comment about the horizontal plane with an accurate hand gesture in which the palm is held flat, face down, and parallel to the floor—also referred to as a “flat-hand” gesture in other gestural typologies (Van Boening and Riggs 2020). However, it is also possible that—while talking about the horizontal plane—the instructor might gesture inaccurately by holding a flattened palm at an oblique angle. 

To foreshadow the findings of Study 1, we did indeed find instances in which people spontaneously made non-horizontal gestures while discussing a horizontal feature or process during an explanation of strike. In Study 2, therefore, we tested the impact of the presence and accuracy of an instructor’s gestures on students’ learning. More specifically, we randomly assigned students to watch one of three instructional videos in which verbal explanations of strikes were accompanied by horizontal gestures, non-horizontal gestures, or no iconic gestures. Students were then asked to complete a strike-mapping task as a measure of their learning. Details about the rationale, methods, and results of the two studies are provided in turn below.

## 2. Study 1

Study 1 was designed to learn whether people—while explaining something about strike, which draws on the concept of horizontality—sometimes use gestures that are held or moved in a non-horizontal position. To address this question, we used archival video data from a study mentioned earlier (Liben et al. 2010). In that study, participants were asked to read a lesson about strike and dip, complete laboratory strike and dip tasks, and then make video recordings to explain these geological concepts to another student. For our current work, we searched the archived videotapes for strike-related verbal explanations involving horizontality. When those segments included iconic gestures, the gestures were coded with respect to whether they were or were not horizontal (i.e., were vs. were not parallel to the floor or laboratory table). We were also interested in learning whether non-horizontal gestures—if found at all—would be produced by people with varying levels of geological experience.

The practical reason that it is important to examine the occurrence of inaccurate strike gestures in both novices and experienced individuals derives from the observation that adults who deliver science instruction to students in school settings vary widely with respect to their expertise in various STEM disciplines. Elementary and middle-school science teachers are commonly required to teach a broad range of sciences (PA Dept. of Education 2009), and thus many teachers are only modestly familiar with some of the concepts and skills they are teaching. Among high school teachers, survey data show that science teachers commonly teach outside their bachelor’s degree content specialty area (Hill and Stearns 2015). Even in higher education, first-year graduate students—who are only beginning to acquire solid expertise in their chosen domain—are assigned to teach undergraduates the very concepts and skills that they themselves are only beginning to learn. Thus, for our goals, it was important to examine instructional gesturing by adults across a range of substantive expertise. 

The identified archived dataset previously described was especially useful for our research because its participants varied with respect to their geological expertise. Specifically, the participants in that earlier study included one sample of university students who reported having had no prior instruction about strike and dip (*novices*) and a second sample of university students who reported prior instruction in structural geology (*geo-students*). Both novices and geo-students had been reported to have accompanied their spoken explanations of strike with iconic gestures (Liben et al. 2010).

We thought it likely that novices would make gestural errors both because they had only just been introduced to the geological concept of strike as part of the research study itself and because they were drawn from disparate majors, including those in the humanities, which neither select for nor encourage spatial skills (e.g., Wai et al. 2009). We thought geo-students might not produce non-horizontal gestures for the inverse reasons. That is, these students could be expected to have entered the research protocol with a better-developed entry-level understanding of the strike concept and with higher-level spatial skills given their STEM interests, majors, and coursework (Liben and Titus 2012; Wai et al. 2009). We thus examined the archival dataset to learn whether novices, geo-students, or members of both groups spontaneously produced non-horizontal (inaccurate) as well as horizontal (accurate) gestures in the course of providing verbal explanations of strikes. 

### 2.1. Method

#### 2.1.1. Participants 

As explained earlier, the original study from which we drew archived video data included two groups of university students. The novice group included 77 undergraduate students who were members of the psychology subject pool of a large, public state university in the northeast United States who had volunteered to participate and who later self-reported no prior instruction (formal or informal) in structural geology. The geo-student group included 21 students who had prior or ongoing instruction in structural geology. Among the latter group were five members of the psychology subject pool who had also volunteered to participate and later self-reported having had prior instruction in structural geology. The remaining 16 members of the geo-student sample included geoscience students attending the same university (11 undergraduate students and five graduate students). These 16 students had responded to announcements made in geology classes; electronic mailings sent to students in geoscience programs; or posters placed on bulletin boards near geoscience classes, facilities, and events. 

Participants recruited via the psychology subject pool received extra course credit for participation; those recruited in other ways received USD 12 for participation. Participants self-reported gender, but no additional demographic information was collected. The university’s student body draws from a large range of urban, rural, and suburban areas; it is largely middle-class and white. Thus, participants in the study had varied socioeconomic and geographic backgrounds representative of the university at large, but the sample was not representative of the United States or global diversity more generally, and thus generalizability is limited.

For the current investigation, we used archived videotaped data from 68 of the original 77 novices (including 36 who self-identified as men and 32 who self-identified as women; *M_age_* [*SD*] = 19.5 [1.4] years). The nine videotapes we excluded came from six participants who failed to explain the concept of strike in their own words (i.e., contrary to instructions, they read directly from the written text of the lessons) and three who omitted an explanation of strike entirely. We used data from all 21 of the geo-students (14 men and 7 women; *M_age_* [*SD*] = 22.7 [3.7] years) because all had explained strikes in their own words when recording their instructional videotapes.

#### 2.1.2. Procedure

After hearing a brief overview of the session and completing the consent protocol, participants read an introductory lesson about strike and dip. The lesson, drawn from a United States Geologic Survey website, has been used in prior research with college students (Liben et al. 2010, 2011) and is reproduced in full in Appendix A.1. After reading the lesson, participants completed model-outcrop strike and dip mapping tasks as an assessment of their mastery of the concepts. Finally, participants were asked to video-record explanations of the two geological concepts for another (unseen) student. While videotaping their explanations, participants retained access to the written lesson (which included poster-sized images of the relevant photographs and maps) and to the tabletop models of outcrops that had been used in the just-completed strike and dip tasks (described further in Study 2 and in greater detail in Liben et al. 2010).

#### 2.1.3. Gesture Coding

**Figure 1:**
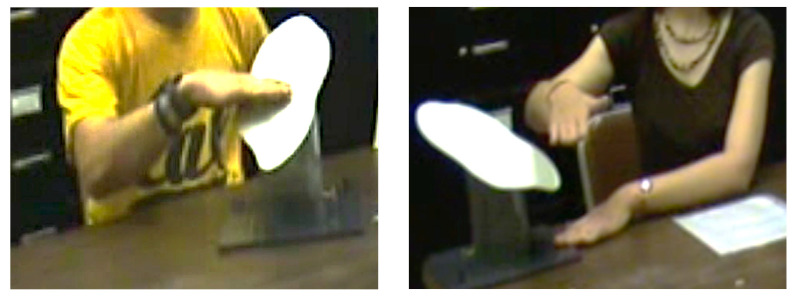
**Illustrative Strike-Related Iconic Gestures.** Example of accurate (**left**) and inaccurate (**right**) strike-related iconic gestures. Each participant produced their gesture while giving a verbal description of a horizontal plane intersecting the face of a model outcrop. Neither participant had been discussing dip while these gestures were made.

An undergraduate research assistant (coder #1) examined video recordings to find all instances in which a participant had provided a strike-related verbal explanation that involved horizontality and was accompanied by an iconic gesture—referred to hereafter in this article as *strike-related iconic* gestures. After all such video events had been found, coder #1 judged each strike-related iconic gesture for its accuracy with respect to horizontality. To be coded as accurate, the angle at which the hand or arm was held (for static gestures) or moved (for dynamic gestures) had to be judged as falling within 20° of horizontal. Gestures judged as deviating by more than 20° off the horizontal were coded as inaccurate. The 20° criterion for categorizing gestures as inaccurate was based on earlier research by Howard (1978) that demonstrated that even adults who fail the traditional test of horizontality (the water level task, described in Study 2) reported waterlines to be unnatural if they appeared to be inclined by more than 15° from the horizontal (an appearance Howard conveyed to participants via trick photography). Illustrations of accurate and inaccurate strike-related iconic gestures are provided in Figure 1.

Each of the strike-related iconic gestures was also coded independently by one of two additional undergraduate research assistants (coders #2 and #3). Thus, each identified strike-related iconic gesture was independently coded for accuracy by coder pairs #1 and #2 or #1 and #3. A random sample of the videos from 20% of the participants was selected to assess their final reliability. Given the relatively small corpus of strike-related iconic gestures, reliability was calculated as percent agreement (see Viera and Garrett 2005), showing 90% agreement. Final categorizations of any strike-related iconic gestures that had received discrepant codes (i.e., gestures that were categorized by one coder in the pair as “accurate” and by the other coder as “inaccurate”) were resolved via discussion among the first author and all three coders. 

### 2.2. Results

#### 2.2.1. Overview

Consistent with the observations reported by Liben et al. (2010), we observed that both novices and geo-students produced iconic gestures during their verbal explanations; that some of these gestures were made in open space surrounding the participant’s body and others were made on or next to a fixed surface (i.e., on a laboratory table or poster); and that some were static (i.e., held in a single, fixed position) and others were dynamic (i.e., moved in some way, as when a participant swept their palm back-and-forth along a horizontal plane). Our new coding was designed to examine the appearance of horizontal versus non-horizontal strike-related iconic gestures in one or both participant groups. Descriptive data on such gestures are provided below, first for the novices and then for the geo-students.

#### 2.2.2. Novices

**Figure 2:**
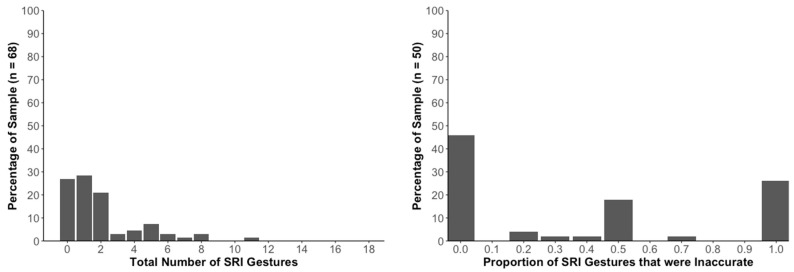
**Incidence and Types of Strike-Related Iconic Gestures in Novices.** The graph on the left shows data for 68 novices. The bars show percentages of this sample that spontaneously produced various numbers of strike-related iconic [SRI] gestures. As conveyed by the figure and noted in the text, most novices produced two or fewer such gestures. The graph on the right shows data from the 50 novices who produced at least one SRI gesture. The bars provide data on what proportions of these SRI gestures were incorrect (non-horizontal).

The left bar graph in Figure 2 shows that in the novice sample, there were between 0 and 11 strike-related iconic gestures per participant. Most participants produced very few such gestures (i.e., 0, 1, or 2 gestures) so that the distribution was positively skewed. On average, novices produced 1.96 gestures (*SD* = 2.23); skew = 1.79; and kurtosis = 3.64.

Among the 68 novices, 50 (74%) produced at least one strike-related iconic gesture. The total number of these gestures produced by the novices was 131, of which 84 (64%) were coded as horizontal or accurate and 47 (36%) were coded as non-horizontal or inaccurate based on the 20° criterion. The right bar graph in Figure 2 shows the percentage of the sample producing various proportions of strike-related iconic gestures that were inaccurate. 

#### 2.2.3. Geo-Students

**Figure 3:**
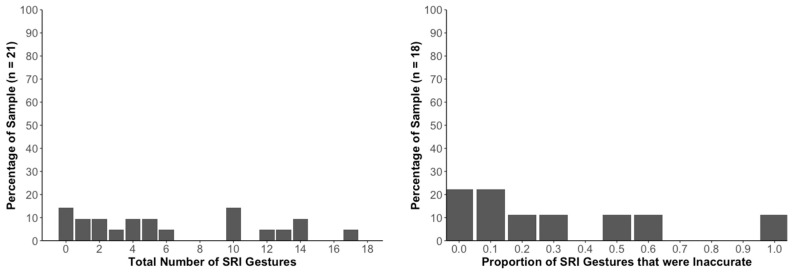
**Incidence and Types of Strike-Related Iconic Gestures in Geo-Students**. The graph on the left shows data for all 21 geo-students. The bars show percentages of this sample that spontaneously produced various numbers of strike-related iconic [SRI] gestures. As shown in the figure and noted in the text, SRI gestures ranged between 0 and 17. The graph on the right shows data from the 18 geo-students who produced at least one SRI gesture. The bars provide data on what proportions of these SRI gestures were incorrect (non-horizontal).

The histograms in Figure 3 show comparable data for the geo-student sample. The left histogram shows a wide range of strike-related iconic gesture frequencies (between 0 and 17 gestures by individual participants). In this sample, the average number of strike-related iconic gestures produced per student was 6.38 (*SD* = 5.39); skew = 0.50; and kurtosis = −1.09.

Among the 21 geo-students, 18 (86%) produced at least one iconic horizontal gesture. The total number of iconic horizontal gestures in the geo-student group was 134, of which 109 (81%) were accurate and 25 (19%) were inaccurate. The right bar graph in Figure 3 shows the percentage of the sample producing various proportions of strike-related iconic gestures that were inaccurate.

### 2.3. Discussion

As explained in the Introduction, the video data analyzed here had originally been examined by Liben et al. (2010) to learn when, where, and how often novices and geo-students spontaneously produced iconic gestures when explaining strike and dip to another person. The current study focused on a subset of these gestures—strike-related iconic gestures. Our new data reveal that the majority of participants in both samples (novices and geo-students) spontaneously produced at least one such gesture. As seen in the histograms in Figure 2 and Figure 3, there was considerable variability within each of the two participant samples in the number of strike-related iconic gestures that were produced.

Our yet more specific focus was on the accuracy of those iconic gestures, a quality that, to our knowledge, had not previously been examined systematically. In both participant groups, some of the strike-related iconic gestures deviated considerably from horizontal. It is particularly interesting that inaccurate gestures were produced not only by novices but also by geo-students. It seems unlikely that students in the latter group would have been struggling to understand the geological concept of strike itself. Furthermore, most students in the geo-student group would be expected to have entered the study with skill in identifying horizontals given past evidence of geo-scientists’ strong spatial skills in general (e.g., Kastens and Ishikawa 2006; Liben and Titus 2012) and strong performance on horizontality tasks in particular (a finding reported by Liben et al. 2010 for this participant sample). At the same time, the task of explaining strikes can be difficult even for highly experienced geology instructors (Liben et al. 2011). Because the findings from Study 1 provided evidence that inaccurate strike-related iconic gestures are generated by people across a wide range of substantive expertise, our next step was to ask if inaccurate gestures like those observed in Study 1 would be detrimental to student learning. If non-horizontal strike-related iconic gestures made by instructors are found to interfere with students’ understanding of geological strike, it would imply that it would be unwise in the absence of further guidance to simply tell instructors to gesture. Instructors—whether having disciplinary expertise or not—might need coaching and practice (e.g., in preservice or in-service workshops) about the use and form of gestures in their teaching. 

## 3. Study 2

In brief, the key goal of Study 2 was to provide an experimental test of whether student performance on a measure of strike understanding would be affected by variations in the inclusion and accuracy of strike-related instructional gestures. All participants were undergraduate college students who reported no prior instruction (formal or informal) in structural geology. Each participant was randomly assigned to view one of three instructional videotaped lessons on strike and dip. In all three versions, the same instructor provided identical verbal explanations of the concepts. Gestures, however, differed across instructional conditions: in one version, the instructor accompanied her verbal strike explanations with accurate (horizontal) gestures; in a second, she included inaccurate (non-horizontal) gestures; and in the third, she omitted iconic gestures entirely.

We predicted that, overall, participants who were randomly assigned to the accurate-gesture instructional video would have a better understanding of strike as indicated by better performance on a subsequent strike assessment task than would participants who were assigned to either of the other two instructional conditions. We further expected that if there were differences between the latter two groups, participants’ strike performance would be lower in the inaccurate-gesture condition than in the no-gesture condition because the former could be expected to interfere with an understanding of horizontality, whereas the latter would simply fail to reinforce the verbal explanation included in the spoken text of the lesson.

As reviewed briefly in the introduction, prior research has shown that higher spatial skills are associated with better STEM performance in general (e.g., National Research Council 2006; Wai et al. 2009) and with better geology performance in particular (Atit et al. 2020; Liben and Titus 2012; Ormand et al. 2014; Shipley et al. 2013; Titus and Horsman 2009). One category of spatial skill—*spatial perception*—requires respondents to process (e.g., observe, represent, or remember) something that is embedded within a potentially distracting context or frame of reference (e.g., finding a simple geometric figure or line hidden within a larger and more complex figure, Witkin and Goodenough 1981). Finding and depicting the strike of a rock outcrop may be expected to use this kind of spatial skill, and indeed, better spatial perceptual skills have been shown to predict better performance on geological-strike tests (Liben et al. 2010, 2011). Additionally, spatial-perception skills have been associated with better map skills (e.g., Liben et al. 2008), a finding that is also relevant to the current study because the task used to assess participants’ strike understanding required participants to draw strike lines on a paper map. Thus, our secondary goal in Study 2 was to determine whether participants’ performance on a test of strike understanding administered after the geology lessons would be moderated by participants’ entry-level spatial perception skills.

The spatial-perception task used in the current study was the water level task (WLT). The WLT was originally developed by Piaget and Inhelder (1956) to assess Euclidean spatial concepts in young children, but subsequent investigators have found the task to be challenging for many adults as well (e.g., Liben 1991; Sholl 1989; Thomas et al. 1973; Vasta and Liben 1996). We selected the WLT for several reasons. First, based on meta- and conceptual analyses of assessments used to assess spatial skills, Linn and Petersen (1985) identified the WLT as an assessment of spatial perception. 

Second, the WLT measures perception of horizontality within conflicting frames of reference, which we hypothesized to be a skill important for understanding geological strikes. As seen in the dictionary definition of strike quoted earlier, a starting point for determining the orientation of strike is finding “the line formed by the intersection of a fault, bed, or other planar feature and a horizontal plane”. That intersection is, by definition, parallel to the ground, but the shape and tilt of the outcrop itself and, later, the shape and orientation of the base map on which the strike line is drawn, provide potentially competing and distracting frames of reference. These may compete with the horizontal and vertical referents available in the surrounding room (e.g., the horizontals of the floor and table; the vertical lines of the door or intersecting walls). For example, a strike line drawn or imagined on the face of the rock outcrop is unlikely to parallel a major axis of the outcrop; a strike line drawn on a map will not parallel the bottom of the page except in particular circumstances (as when strike is 090° and the map is north-facing (i.e., north is at the top of the paper)).

Finally, the WLT has practical advantages because it is an unusually brief spatial assessment, and irrespective of age or performance levels, respondents typically enjoy completing the task. 

To explore whether the impact of the instructional (gesture) condition on mastery of the geology lesson was moderated by participants’ spatial-perception skills, we thus tested participants’ performance on spatial perception via the WLT. To better isolate the unique effect of spatial perception, we also measured and controlled for performance on tests of two other spatial skill categories identified by Linn and Petersen (1985)—*spatial visualization* and *mental rotation.* In the terminology used by Linn and Petersen (1985), spatial visualization is a skill in understanding the effects of multistep spatial manipulations. An illustrative assessment is a task in which a piece of paper is depicted first as undergoing a series of folds, then as a hole punched in various locations, and finally as unfolded. The respondent is asked to select which of several choices shows how the paper would look after being unfolded. Mental rotation is the skill of imagining rotations of two-dimensional [2D] figures or three-dimensional [3D] objects. An illustrative assessment is a task in which respondents are asked to predict what an array of blocks would look like after being moved in specified ways along the X, Y, and Z axes (e.g., as indicated by arrows). 

Based on our hypothesis that a participant’s spatial-perception skill would play a particularly important role in completing a geological strike task, we predicted that performance on the WLT would predict participants’ performance on a test of strike understanding, even after removing variance accounted for by tests of the other two categories of spatial skills (i.e., spatial visualization and mental rotation). 

Past research has already demonstrated links between participants’ WLT scores and how well they perform on a test of strike understanding following a brief geology lesson (Liben et al. 2011). The new question addressed here was whether participants’ entry-level spatial-perception skills would moderate the impact of instructional-gesture conditions (accurate, inaccurate, or no gestures) on strike learning. Our basic a priori hypothesis was that there would be different patterns of gestural-condition effects across the participant groups. For reasons explained next, we had several alternative ideas about exactly how participants’ spatial-perception skills might interact with gestural-instructional conditions. 

We thought it possible that students with high spatial-perception skills would find accurate gestures unnecessary and might simply ignore inaccurate gestures. Alternatively, students with high spatial-perception skills might be especially likely to notice the non-horizontality of inaccurate gestures and thus become confused by the conflict between verbal statements about identifying the intersection of a horizontal plane and the surface of a geological structure. In the former case, participants with high spatial-perceptual skills could be expected to perform similarly across all three conditions; in the latter, they could be expected to perform worst in the inaccurate-gesture condition. We could likewise imagine competing predictions for students with low spatial-perception skills. We thus conducted analyses on the interaction between spatial skills and instructional conditions without a priori hypotheses.

Before leaving the topic of individual participants’ characteristics, we note that researchers, educators, and policy leaders often report that STEM engagement and success differ by participant gender. It is, however, misleading to make sweeping generalizations about gender and STEM because gender distributions differ dramatically across specific STEM scientific disciplines (e.g., see Leaper 2015), and many gender differences evaporate entirely after controlling for variance in relevant cognitive skills such as spatial skill or experience such as computer use (e.g., Hyde 2005; Jirout and Newcombe 2015; Lauer et al. 2019; Liben et al. 2008). Given limitations in statistical power, we limited our analyses to the two factors of primary interest—that is, experimental assignment to an instructional (gesture) condition and pre-existing individual differences in spatial skills. 

### 3.1. Method

#### 3.1.1. Participants and Procedure Overview

A new sample of undergraduates who did not have prior experience in geology was recruited from a student population like that reported in Study 1, using comparable recruitment procedures. The resulting sample contained 167 students who self-reported as men (n = 84) or women (n = 83); *M_age_* [*SD*] = 18.9 [1.1] years. Assignments to instructional conditions were made in a rotating fashion within gender to achieve accurate (n = 56), inaccurate (n = 56), and no-gesture (n = 55) instructional conditions. A research assistant worked with each participant individually. After administering the informed consent protocol, the assistant administered four phases of the procedure proper. In the first phase, the participant was given the only untimed spatial test, specifically the water level task (WLT). Phase two was the presentation of the instructional video (shown twice). In phase three, the research assistant administered a measure of geology learning—the strike-mapping task. The final phase included three timed spatial tests selected to measure spatial visualization, 2D mental rotation, and 3D mental rotation. Below, we begin by providing details about the instructional lesson and then describe the measure of geology learning; we close by describing the four spatial tasks. 

#### 3.1.2. Instructional Procedure and Assessment 

**Instructional Geology Video and Stimulus Materials**. Participants were told they were going to watch an instructional video on the geological concepts of strike and dip and that it would be repeated a second time. Before showing the video, the experimenter explained to participants that they would later be completing tasks based on what they learned from the video lesson. The script for the instructional video (see Appendix A.1) was adapted from the written lesson that had been used by the investigators of Study 1 (see Liben et al. 2011). During the video-recorded lesson, the instructor referred to photographs and diagrams pasted on a poster board. The participant was also given a booklet with smaller versions of the photographs and diagrams that were shown in the poster (see Appendix A.2) so that images were available for the participant to consult as desired during the lesson. The booklet of images and figures was removed immediately before the participant was given the strike-mapping task (described below). 

The iconic gestures accompanying a verbal explanation of strike included in the instructional videos varied with respect to gesture locus, action, and accuracy, thereby drawing on the kinds of variations in iconic gestures that had been produced spontaneously by participants in Study 1. Specifically, we included static and dynamic gestures that were made in open spaces surrounding the instructor’s body as well as on fixed surfaces such as the table surface or a photograph mounted on the poster. 

Three versions of the lessons were recorded. In brief, the videotaped lessons differed across instructional conditions with respect to the presence and accuracy of strike-relevant iconic gestures. During the lesson, the instructor pointed to the supplemental pictures and figures when the script referred to those figures and—in the accurate and inaccurate gesture conditions—made gestures when the script covered concepts referring to horizontality. For example, the first sentence of the instructional script was “Many kinds of rocks form in broad, ***flat layers***, which ***stack up like a pile of books*** or the ***layers of a cake***”. The phrases that are italicized and bolded here indicate where gestures accompanied the verbal description. 

**Figure 4:**
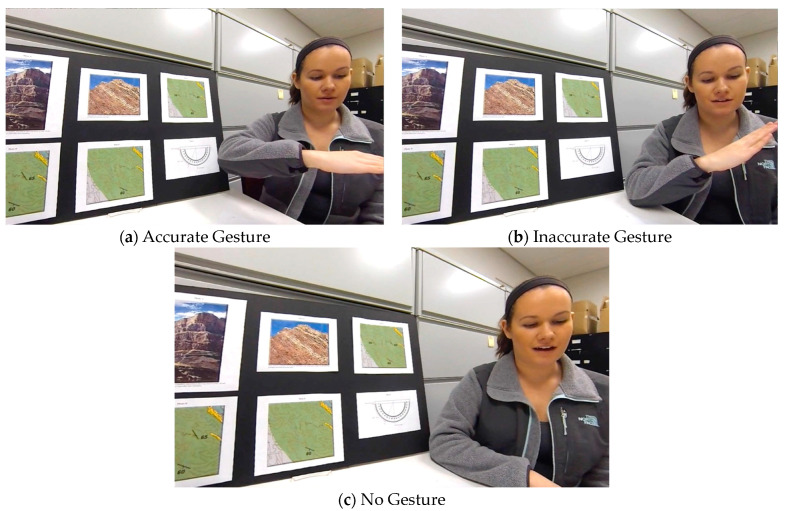
**Examples of Instructional Video Conditions: Accurate, Inaccurate, and No Gesture.** Snapshots from the three instructional videos at the identical point in the script.

In the accurate-gesture instructional video, the instructor included static and dynamic horizontal gestures while delivering the relevant parts of the instructional script (see Figure 4a). For example, the gesture made during the phrase “flat layers” involved a static, horizontal hand and arm orientation, reinforcing the verbal description of something that is flat or horizontal. The gesture during the phrase “stack up like a pile of books” involved a dynamic, horizontal hand orientation and horizontal motion, thus providing a gestural representation of an increasingly tall stack of books lying flat. The gesture during the phrase “layers of a cake” also showed a series of dynamic, horizontal hand orientations placed over one another, thus “painting” a gestural image to represent horizontal layers of a cake.

In the inaccurate-gesture instructional video, the instructor also accompanied her verbal descriptions with static and dynamic gestures like the ones described above, but in this condition, the instructor’s gestures were not, in fact, horizontal (see Figure 4b). That is, the instructor made non-horizontal gestures when talking about concepts that referred to horizontality. In the above example, instead of holding and moving her hand in a horizontal position, the instructor held her hand at a slant and made slanted motion gestures so that the gestural representations did not convey the meaning of the words.

In the no-gesture instructional video, the instructor avoided iconic gestures entirely. 

In all three instructional conditions, the instructor made deictic (attentional) gestures by pointing to supplemental pictures and figures when referenced in the script (e.g., pointing to Photo A when it was mentioned). Across conditions, the instructor presented the material at the same slow but natural pace, so that all three versions were approximately 4 min long. As forewarned, each participant was shown the identical instructional video twice.

**Student Learning: Strike-Mapping Task.** After watching the video lesson for the second time, participants were asked to complete a strike-mapping task like the one that had been administered in the earlier study by Liben and colleagues (2010). The task was given in a room (11′ × 19′) in the psychology department. The participants were seated at the long edge of a rectangular table; an amorphous-shaped model outcrop was placed on the table in eight predetermined locations and orientations, randomly ordered across participants. After each new placement of the model, the participant was given a new copy of a black-and-white plan view map of the room, which depicted the room’s walls, doors, and large furniture (including a bank of vertical file cabinets, two banks of lateral file cabinets, and a computer desk). Omitted from the map was an outline of the table at which the participant was seated. This omission was intended to avoid providing an immediate rectangular frame of reference for the outcrop and strike line so that the lab-based mapping test would more closely resemble what a geologist does when recording strike lines on maps of larger and less differentiated outdoor environments.

For each of the eight placements of the model outcrop, the participant was asked to draw the strike line on the room map. The dependent variable was a degrees-error measure, obtained by measuring how many degrees the participant’s drawn strike line deviated from the correct orientation of the strike line. Each deviation error could thus range between 0° and 90°. Deviations were averaged across the eight items, so higher scores indicated worse performance.^1^

#### 3.1.3. Spatial Skill Test Battery 

**Spatial Perception.** We used a 6-bottle version of the water level task (WLT; Liben and Titus 2012) to measure spatial-perception skills. This task includes line drawings of straight-sided bottles tipped 30°, 45°, and 60° to the right and left and asks participants to draw a line inside each bottle to show “where the water would be if the bottle were about half full and held in the position shown”. Lines within 5° of the horizontal were categorized as correct. As in earlier research (e.g., Liben 1978; Thomas and Lohaus 1993), participants with zero or one error were categorized in the high-water level group (high-WLG: 37 men; 16 women); those with two or more errors were categorized in the low-water level group (low-WLG: 47 men; 67 women). The gender distribution observed here is similar to distributions found in previous studies with similar participant samples (e.g., Sholl and Liben 1995).

**Spatial Skill Covariates**. To identify the unique contribution of participants’ spatial perception to performance on the strike-mapping task, we also administered tests drawn from the two other categories of spatial skills identified by Linn and Petersen (1985). To tap spatial visualization, we used a modified version of the Educational Testing Service paper-folding test (PFT; Ekstrom et al. 1976). The version we used included 20 items, each containing two, three, or four drawings depicting a sheet of paper that was folded one or more times. The drawing that showed the final fold was followed by a drawing that included circles to show places where the folded layers of paper were then hole punched. For each item, respondents were then asked to select which of five drawings showed where the holes would be once the paper was again unfolded. Scores were the number of correct responses marked within 5 min, minus one-fourth the number of incorrect responses marked (i.e., deductions were made for incorrect responses but not for skipped items). Cronbach’s α in the current data set was .67.

Mental rotation skills were assessed using both two- and three-dimensional (2D and 3D) mental rotation (MR) tasks. The 2D-MR task was a modified version of the spatial relations subtest from the primary mental abilities test (Thurstone 1962). In each of the 30 rows, a simple line drawing (model) was shown at the far left, followed by five additional figures. Participants were given 2 min to mark all figures that showed the model rotated in the plane, but to refrain from marking any figures that showed the model flipped over (irrespective of its rotation in the plane). Scores were the number of rotated figures that were correctly marked, minus the number of flipped-over images that were incorrectly marked (Cronbach’s α = .85). The 3D-MR task was a shortened version of the Purdue Visualization of Rotations Test (Bodner and Guay 1997), in which participants were allotted 8 min to respond to 10 items. Each item showed two drawings of the same solid object, the second of which depicted the object after it had been rotated in three-dimensional space. The participant was asked to apply the same rotation strategy to a drawing of a different solid object and select, from five drawings, the one showing how that rotated object would look. The score was the number of correct selections, thus allowing scores to range between 0 and 10 (Cronbach’s α = .71).

### 3.2. Results

#### 3.2.1. Overview

The analysis was designed to examine participants’ mastery of the information about strikes that had been covered in the instructional videos and to determine if this mastery differed systematically in relation to the experimentally manipulated assignment to the instructional condition. As discussed in the introduction to Study 2, we also designed the study to explore whether the effects of the instructional condition were moderated by participants’ spatial-perception skills. The latter was examined by including in our analyses a between-subject participant variable of perceptual skill level, operationalized as being categorized into either the low-WLG or the high-WLG.

As described in the Student Learning: Strike-Mapping Task sub-section of the Method, the dependent measure we used for the analyses reported here is the continuous measure of strike-line error, so that lower scores indicate better performance. A 3 (Instructional Condition: accurate, inaccurate, no gesture) X 2 (Spatial-Perception Skill: low-WLG vs. high-WLG) ANCOVA was conducted to explore the potential interaction between the instructional condition and participants’ spatial-perception skills while controlling for spatial visualization skill (PFT scores) and mental rotation skills (2D- and 3D-MR task scores). In support of the decision to include these three spatial task scores as covariates is the finding that all three are negatively correlated with strike-mapping task error. In other words, as spatial skill increases, error on the strike-mapping task decreases, as shown in Table 1. As an alternative analytic approach, we also conducted multiple linear regressions using a continuous measure of water-level task performance, dummy-coded condition variables, and two-way interaction terms. The regression results were identical to the ANCOVA, and we thus report the latter, which eases communication of group comparisons. All effect-size magnitudes are reported using classifications provided by Cohen (1988).

#### 3.2.2. Results on Strike-Mapping Task: Interaction between Gestural Condition and Participants’ Spatial-Perception Skill

**Figure 5:**
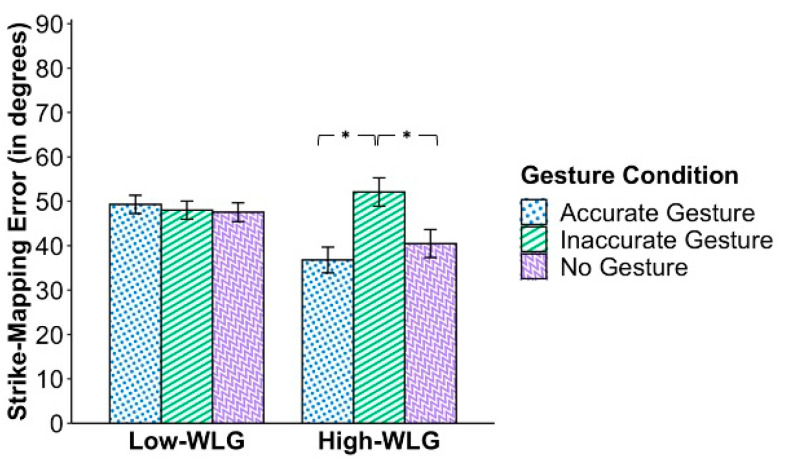
**Mean Strike-Mapping Task Error by Water Level Group (WLG) and Gesture Condition.** Interaction between water level group (low or high) and gesture condition. Strike-mapping error is the mean degree error, so lower scores indicate better performance. Estimated marginal means are presented, controlling for performance on the paper-folding test, the 2D mental rotation task, and the 3D mental rotation task. Error bars indicate standard error. * *p* < .05.

As predicted, analyses of performance on the strike-mapping task revealed a significant two-way interaction between instructional condition and spatial-perception skill, *F*(2,156) = 5.40, *p* = .005, partial η^2^ = .07 (medium effect size), shown graphically in Figure 5. Simple effects tests indicated that for the low-WLG, there was no significant impact of the instructional condition (*p* = .885). For the high-WLG, there was a significant main effect of condition, *F*(2,46) = 5.11, *p* = .010, partial η^2^ = .18 (large effect size). Pairwise comparisons indicated that among the participants with high spatial-perception skill (high-WLG participants), those assigned to the inaccurate-gesture condition had significantly larger errors (i.e., worse performance) on the strike-mapping task than did high-WLG participants who had been assigned to either the accurate or no gesture conditions, *M_diff_* = 15.99°, *SE* = 5.07, *p* = .003; *M_diff_* = 10.92°, *SE* = 5.25, *p* = .043, respectively. There was no significant difference between performance in the accurate versus the no gesture conditions (*p* = .324).

#### 3.2.3. Results on the Strike-Mapping Task: Subsumed Main Effects of Gestural Condition and Participants’ Spatial-Perceptual Skill 

Even though main effects are subsumed under the significant two-way interaction just described, we next report main effects. In part, we do so to provide a complete report. More importantly, however, we do so because instructional curricula for students and professional development for teachers must often be delivered to entire classrooms of students or entire groups of teachers at once, rather than to sub-groups of people who have differing levels of relevant skills or knowledge as assessed by relevant measures (e.g., tests of entry-level spatial perceptual skill or content expertise). 

Returning to the analysis of strike-line error as the measure of participants’ success in learning the concept of strike from the geology lesson, there were significant main effects of instructional condition, *F*(2,156) = 4.30, *p* = .015, partial η^2^ = .05 (medium effect size), and of spatial-perception skill, *F*(1,156) = 4.88, *p* = .029, partial η^2^ = .03 (small-to-medium effect size). 

With respect to instructional condition, pairwise comparisons show that participants in the inaccurate-gesture condition made larger errors (*M* = 50.05°, SE = 1.87) than did participants in either (a) the no-gesture condition (*M* = 44.01°, SE = 1.83), *p* = .022, or in (b) the accurate-gesture condition (*M* = 43.04°, SE = 1.77), *p* = .007. There was no significant difference in error sizes between the latter two conditions, that is, between the no-gesture and the accurate-gesture conditions, *p* = .701. 

With respect to levels of participants’ spatial skills, pairwise comparisons showed that those with higher spatial-perception skills made smaller errors (high-WLG: *M* = 43.12°, SE = 1.87) than did those with lower spatial-perception skills (low-WLG: *M* = 48.27°, SE = 1.22). Thus, if differences in individual learners’ spatial-perception skills must be ignored, results suggest that what would be of greatest importance is to avoid inaccurate gestures during instruction. 

### 3.3. Discussion

Participants in Study 2 who viewed instructional videos about the geological construct of strike made smaller errors overall on assessments of their strike understanding if they had entered the study with higher rather than lower spatial-perception skills (operationalized as being in the high- rather than the low-WLG). This finding is consistent with findings from earlier correlational research, which found a significant association between higher spatial skills and better performance on strike-mapping tasks given in both large, natural outdoor spaces and in smaller indoor laboratory spaces (Liben et al. 2010, 2011).

Extending beyond prior research, in Study 2, we investigated the impact of different kinds of gestures during a videotaped lesson about strike and dip. This manipulation allowed us to test whether students’ understanding of strikes (as assessed by performance on the strike-mapping task) was affected by the inclusion and accuracy of instructional gestures. Because we assessed individual participants’ spatial skills, the study also enabled us to test whether differences in students’ entry-level spatial perception skills would moderate the impact of gestural conditions on learning. As hypothesized, the findings did indeed show that the impact of gestural conditions varied in interaction with participants’ entry-level spatial perception skills. 

As seen in Figure 5, students with relatively poor spatial perceptual skills (i.e., low-WLG participants) showed no significant differences in learning outcomes as a function of the instructional condition to which they had been assigned. That is, low-WLG participants who received the instructions with accompanying strike-related iconic gestures performed no better on the strike-mapping task than did low-WLG participants who had received the instructional video with no strike-related iconic gestures at all. Similarly, low-WLG participants were not harmed by seeing non-horizontal gestures in the inaccurate-gesture condition. One might infer that students who have low spatial-perception skills were, by and large, oblivious to spatial information provided by the instructor’s gestures during the videotaped lesson. Indeed, this inference is perhaps directly implied in the way that Linn and Petersen (1985) defined spatial perception in the first place, that is, as a skill that allows individuals to perceive spatial features or patterns contained within complex, distracting, and even conflicting contexts. When considered in the context of the current study, this definition might suggest that participants with limited spatial-perception skills are unlikely to notice the angle at which a hand or arm was held or moved amid the cacophony of words, diagrams, photographs, and other movements included in the video lesson. If true, it would not be surprising that students in the low-WLG neither profited from accurate gestures nor suffered from inaccurate ones.

Although the mean degree error might suggest that the low-WLG participants had been drawing lines randomly (the group’s mean deviation approximated the midpoint of possible deviations), the standard errors shown in Figure 5 and findings from the number-correct measure (see Footnote #1) clearly indicate a range of performance in both participant groups. Future studies with far larger samples are needed to conduct latent class analyses to identify sub-groups of participants who might be using different strategies. For example, some response patterns may suggest random performance; others may suggest the use of a systematic (albeit problematic) strategy, for example, drawing lines to indicate the direction of the long axis of the face of the model outcrop. Perhaps different strategies could be linked to participant variables not yet addressed, for example, participants’ differential prior experience in using topographic maps or differential experience in environments that vary in the number and types of outcrops (e.g., mountainous areas of the Western US vs. flat grassland areas of the Midwestern US).

The pattern of findings among the students with high entry-level spatial-perception skills is noticeably different insofar as learning outcomes did show a significant effect of instructional conditions in the high-WLG. Specifically, although high-WLG participants—like low-WLG participants—did no better on the strike-mapping task from a lesson that added accurate gestures to otherwise entirely verbal instructions (i.e., neither participant group showed better performance in the accurate- than in the no-gesture condition), high-WLG participants performed significantly worse if they had been assigned to the inaccurate-gesture condition. This finding implies that the high-WLG participants in some way must have processed (and, we surmise, been confused by) the mismatch between the instructor’s words (“horizontal”) and her gestural representations (i.e., her non-horizontal gestures).

In sum, the data from the current study provide no evidence of a salutary effect of adding accurate (horizontal) gestures to instruction about the geological concept of strike. This finding held for participants irrespective of their spatial perceptual skill. The data do, however, provide evidence that inaccurate gestures can hinder student learning, an effect that is accounted for by the negative impact of inaccurate gestures on students who entered the study with high spatial-perception skills. Presumably, these students’ sensitivity to perceiving spatial qualities of stimuli makes them vulnerable to being confused when words convey “horizontality,” but the accompanying gestures do not. 

## 4. General Discussion

### 4.1. Highlights of the Current Work

To summarize, past research in developmental, cognitive, educational, and learning sciences has demonstrated many challenges as well as many opportunities for STEM teaching and learning. As reviewed in the introduction, iconic gestures are often used while thinking about, teaching about, or otherwise communicating about STEM concepts or phenomena. Correlational data have shown significant associations among individuals’ domain expertise, spatial skills, and the production of iconic gestures. 

Building on these correlational findings, researchers and educators have suggested that greater use of iconic gestures in STEM teaching and learning might be valuable. For example, within the domain of geology—the illustrative STEM discipline addressed in the current research—instructors have been encouraged to incorporate more gesture in their teaching (e.g., Kastens et al. 2008; Roth 2001). There has, however, been relatively little research testing the impact of such gestures using experimental research designs—that is, by assigning instructional conditions randomly and assessing students’ learning. Furthermore, to our knowledge, there has been no systematic study of whether instructors’ iconic gestures are sometimes misleading, perhaps confusing, rather than benefiting learners. 

Study 1 of our current research was designed to learn whether people who are trying to explain the geological concept of strike to novices (i.e., those not already knowledgeable about structural geology) spontaneously produce inaccurate iconic gestures as part of their strike explanations. The findings from Study 1 showed that, indeed, some instructor-produced strike-related iconic gestures involving horizontality were not horizontal. Importantly, inaccurate gestures like these were produced not only by geology novices but also by those who had entered the study after having had previous or ongoing instruction about structural geology.

We designed Study 2 to investigate whether the qualities of iconic gestures (present vs. absent; accurate vs. inaccurate) impact students’ learning. Specifically, students without prior instruction in structural geology were randomly assigned to watch one of three video-recorded lessons, all delivered by the same person following the identical verbal script. Across conditions, strike-related explanations were accompanied by iconic gestures that were horizontal (the accurate condition), non-horizontal (the inaccurate condition), or by no iconic gestures at all (the no-gesture condition). As reviewed in the introduction, because students’ spatial skills have been shown to correlate with science learning—including the learning of geological concepts—we also designed Study 2 to study whether the impact of instructional conditions would be moderated by participants’ spatial-perception skills. As predicted and as detailed in the Results section of Study 2, the impact of instructional conditions was indeed moderated by participants’ spatial-perception skills, even after controlling for performance on measures of two other major categories of spatial skills.

Interestingly, the addition of accurate gestures to the videotaped lesson led to no significant advantage on the strike-mapping task for either group of participants, one composed of students who had higher spatial-perception skills (the high-WLG) and the other composed of students with lower spatial-perceptual skills (the low-WLG). That is, in neither participant group was the mean error on the strike-mapping task significantly lower in the accurate-gesture condition than in the no-gesture condition. 

The addition of inaccurate gestures, however, affected the two participant groups differently. Among low-WLG participants, strike-mapping task performance was no worse as a result of having been assigned to the inaccurate-gesture condition than it had been under the no-gesture or accurate-gesture conditions. In contrast, among the high-WLG participants, the addition of inaccurate gestures during the videotaped lessons was detrimental. That is, performance on the strike-mapping task was significantly worse if students with higher spatial-perception skills had been randomly assigned to the inaccurate-gesture condition than if they had been assigned to either the accurate or the no-gesture instructional conditions. 

As suggested earlier, we infer that students who have high spatial-perception skills are especially susceptible to confusion from inaccurate gestures because they are (by definition) better able to perceive the orientations of the gestures. These participants are therefore more likely to be confused when verbal explanations convey “horizontality” while gestures do not. These high-WLG students would likely find accurate gestures unremarkable and, after initial processing, simply ignore them. However, high-WLG students assigned to inaccurate gestures might instantly realize that something is amiss and give those gestures extra attention. Future research using eye-tracking methodologies might be revealing in this context. We infer the inverse is operating among students with low spatial-perception skills. That is, we infer that participants with limited spatial-perception skills would be unlikely to notice the angle at which an instructor’s hand or arm was held or moved during verbal instruction. Thus, it is not surprising that students with low spatial-perception skills (i.e., low-WLG students) neither benefited from accurate gestures nor suffered from inaccurate ones. However, as noted earlier, additional research is needed to try to identify and explain systematic error patterns. 

### 4.2. Limitations and Future Directions

The research we have reported is, to our knowledge, the first (a) to document adults’ spontaneous use of inaccurate versus accurate gestures in the course of explaining a STEM concept to another person; (b) to employ an experimental design to evaluate learners’ conceptual understanding of the targeted concept in relation to instructional conditions that varied with respect to the presence and accuracy of iconic gestures; and (c) to investigate whether the impact of gestural conditions on student learning is moderated by learners’ entry-level spatial-perception skills. 

As detailed in the results and discussion sections of Studies 1 and 2, and as highlighted in the preceding part of the general discussion, our findings show that gestures can indeed affect how learners perform on a subsequent assessment of their understanding of the targeted STEM concept (here measured by the strike-mapping task). Most important are two findings. First, adding accurate gestures to verbal explanations of strikes gave no demonstrable boost to students’ performance on the strike-mapping task. This was true among learners who were categorized as having low spatial-perception skills as well as among those categorized as having high spatial-perception skills. Second, the addition of inaccurate gestures led to significantly lower levels of performance on the strike-mapping task—with a large effect size—in the sample of students with high spatial-perception skills.

#### 4.2.1. The Need for Direct and Conceptual Replications

It is important to be extremely cautious, however, before using the current findings to argue against the benefit of instructional gestures for STEM teaching. First, it would be important to conduct direct replication research (see, for example, Lindsay 2015). This is especially important here given that, as described in the Introductions to Studies 1 and 2, we did not begin with a complete set of predictions. Thus, it would be useful to conduct additional studies using the same STEM target (geological strike), the same videotaped lessons with the same gestural conditions, the same learning assessment (the strike-mapping task), and the same choice of categories and measures of spatial skills (i.e., the WLT, PFT, 2D-MR, and 3D-MR). If direct replications lead to similar results, additional research should include conceptual replications, perhaps still focusing on the same STEM target of geological strike but using a different measure of strike understanding (e.g., drawing a strike symbol on a map to show the location and orientation of an outcrop found in a field area).

#### 4.2.2. Extensions and Generalizations

Assuming the phenomena reported here are directly replicable, future investigators should conduct conceptual and population extensions designed to address the broader generalizability and utility of the current studies’ rationale and results. For example, one could implement an experimental design similar to the one used in Study 2 but extend the work to encompass a wider range of STEM disciplines and topics. Or, one could focus on a larger (or different) variety of spatial and cognitive skills (e.g., spatial and verbal working memory) as covariates or moderators. For example, research on linguistic and visual processing in adults suggests that better working memory facilitates eye gaze to visually relevant objects (Huettig and Janse 2016). Perhaps individuals with higher verbal working memory are harmed more by inaccurate gestures because they are better equipped to process verbal and visual channels simultaneously. Alternatively, perhaps individuals with more restricted verbal working memory are more susceptible to harm from inaccurate gestures because they routinely rely more on visual than verbal channels. 

Additional research is also needed to examine whether current findings are relevant for populations recruited from non-university settings. The importance of going beyond WEIRD samples (Henrich et al. 2010) to involve culturally, racially, ethnically, linguistically, and socioeconomically diverse populations has become increasingly clear. Diversification and inclusivity are needed to advance understanding of human development and to design successful educational interventions (García Coll and Magnuson 2000). Illustratively, past work has already documented cultural variations in gesture. For example, Richland (2015) found that teachers from Hong Kong and Japan used more gestures to link mathematics concepts than did teachers from the United States. Teachers from Hong Kong and Japan were also more likely than teachers from the US to tailor their gestures to students’ previous experiences.

Another valuable direction for extension concerns variations in instructional modalities such as live versus video-recorded instruction or passive viewing versus active gesturing. In the current study, individuals passively watched a recorded STEM lesson—an instructional format chosen to parallel lectures commonly provided in US K-16 classrooms and in online instruction delivered during the COVID pandemic. Although prior research shows that passive viewing of gestures can improve STEM learning (e.g., Cook et al. 2013; Rueckert et al. 2017), active gesturing appears to be even more effective (Goldin-Meadow et al. 2012).

## 5. Conclusions

In conclusion, the findings from the current study add to previous work in demonstrating the power of iconic gestures for STEM instruction and lead us to suggest several important directions for future research. The findings already in hand, though, are sufficient to lead us to sound a cautionary note: We warn against overarching recommendations that simply urge teachers to include gestures in their STEM instruction. Findings from Study 1 demonstrate that both novices and experienced individuals sometimes use non-horizontal gestures to accompany verbal explanations involving horizontality, although additional research is needed in actual classrooms and field settings to determine how commonly non-horizontal strike-related iconic gestures appear outside laboratory contexts. Findings from Study 2 suggest that at least some students are likely to be confused when they encounter instructions in which gestures are mismatched to the accompanying words. Our findings suggest that in the quest to harness gestures as tools for STEM education, it may be important to design professional development for teachers as well as instructional curricula for students.

## Figures and Tables

**Table 1:** Correlations of Performance on Assessments.

Task	°Error	WLG	2D-MR	3D-MR
1. Strike-Mapping Task (°error) ^a^				
2. Spatial Perception (WLG) ^b^	−.29 **			
3. 2D Mental Rotation (2D-MR)	−.16 *	.24 **		
4. 3D Mental Rotation (3D-MR)	−.16 *	.35 **	.40 **	
5. Spatial Visualization (PFT) ^c^	−.27 **	.38 **	.48 **	.44 **

^a^ The dependent measure is degrees error; thus, higher scores represent worse performance. ^b^ The low-Water Level Group (WLG) is coded 0; the high-WLG is coded 1. ^c^ PFT is the Paper Folding Test. * *p* < .05, ** *p* < .01.

**Figure A1:**
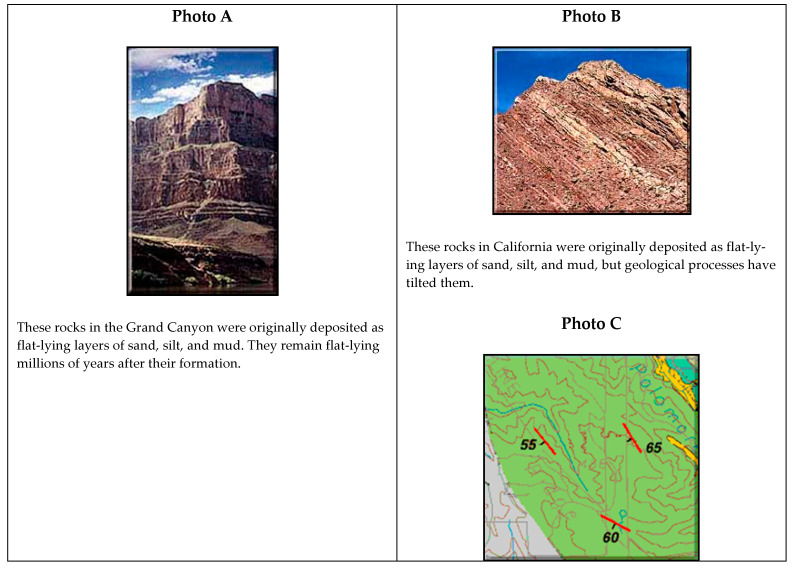

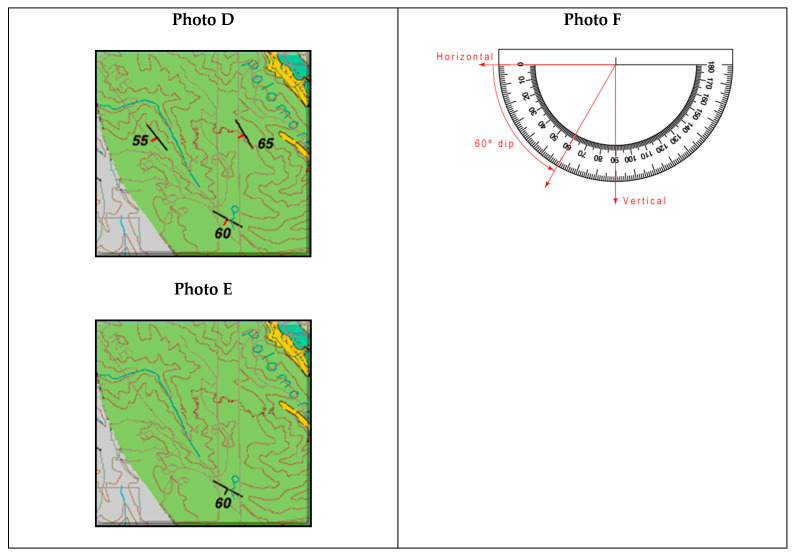
Text and figures based on material from the U.S. Geologic Survey (n.d.) and adapted for use in Liben et al. (2011).

## Data Availability

Please contact Corinne A. Bower (cbower3@calstatela.edu) to request access to the de-identified data.

## Note

1 As an alternative way to examine the data, we also created a number-correct measure. Responses that deviated ≤30° from the correct orientation were coded as correct and awarded a point; other responses received no credit. Total scores ranged between 0 and 8 and were normally distributed for the entire sample. Distributions were normal within each of the water level groups as well, although the range was slightly smaller in the low-WLG (0 to 6 correct) than in the high-WLG (0 to 8 correct). An identical ANCOVA using the correct number as the dependent variable yielded results identical to those found with the average-degrees measure reported above. Findings reported in the text proper are based on the continuous error measure.

## Appendix A

### Appendix A.1. Instructor’s Script for Geology Lesson

Many kinds of rocks form in broad, flat layers, which stack up like a pile of books or the layers of a cake. In some areas, like in Photo A, thick stacks of rock layers that have built up over millions of years remain in their original flat orientation. Now take a few seconds to look at Photo A in your booklet.

In other places, however, the forces that make earthquakes do not leave the beds flat for long but bend or tilt them, as in Photo B. Now take a few seconds to look at Photo B in your booklet.

Tilted rock layers are shown on a geological map with a *strike and dip symbol*. The symbol is placed on the map at the point where a geologist in the field made a measurement of the layer’s tilt.

The symbol consists of three parts: a long line, a short line, and a number, as shown on the map in Photo C. Now take a few seconds to look at Photo C in your booklet.

The long line on the map is called the *strike line*. The strike line is red in Photo C. Now take a few seconds to look at Photo C in your booklet. On any tilted surface, one can always find a direction that is horizontal. Think about walking on the side of a hill; there is always a way to go that is neither up nor down but is level or horizontal. Again, there is always a way to go that is neither up nor down but is level or horizontal. For a tilted rock surface, the direction that is horizontal and is on the rock surface is called the “strike direction”. The strike direction is shown on the map by the long line called the *strike line*.

Remember that a strike and dip symbol consists of three parts: a long line, a short line, and a number, as shown on the map in Photo D. Now take a few seconds to look at Photo D in your booklet.

The short line on the map is called the *dip line* and shows which way the rock layer is tilted. The dip line points in a downward direction. The dip line is red in Photo D. Now take a few seconds to look at Photo D in your booklet.

Remember that a strike and dip symbol consists of three parts: a long line, a short line, and a number, as shown on the map in Photo E. Now take a few seconds to look at Photo E in your booklet.

The number is called the *dip* and shows how much the rock surface is tilted, in degrees, from horizontal. Zero degrees would indicate that the layer is not tilted at all, so that means it is completely flat. Ninety degrees would mean that the layer is vertical—or straight up and down. In the example in Photo E, the layer is titled 60° below the horizon, so we say the “dip is 60°”. Now take a few seconds to look at Photo E in your booklet. Photo F shows a protractor illustrating the 60° dip. Now take a few seconds to look at Photo F in your booklet.
